# A Holistic Approach to Burn Rehabilitation Managed With Split Skin Grafting in an Elderly Woman: A Case Report

**DOI:** 10.7759/cureus.66246

**Published:** 2024-08-05

**Authors:** Mayuri R Zoting, Mansi Subhedar, Subrat N Samal

**Affiliations:** 1 Community Health Physiotherapy, Ravi Nair Physiotherapy College, Datta Meghe Institute of Higher Education and Research, Wardha, IND; 2 Musculoskeletal Physiotherapy, Ravi Nair Physiotherapy College, Datta Meghe Institute of Higher Education and Research, Wardha, IND

**Keywords:** functional mobility, quality of life in the elderly, burn rehabilitation, physiotherapy management, pain management

## Abstract

This study aims to provide a holistic approach to burn rehabilitation that prioritizes patient care, pain management, mental health, and functional improvement. A 71-year-old woman visited the Physiotherapy Outpatient Department with the main concerns of burns on her right hand, limited movement in the fourth and fifth fingers for two months, swelling on her right hand, and wound contracture. She underwent split skin grafting at the hospital. The case study findings demonstrated the effects of physiotherapy interventions on the burn subject, specifically regarding scar formation improvement, functional ability enhancement, and pain reduction. This case study revealed that administering the mentioned workout to the individual proved advantageous, with pain decreasing on the visual analog scale (VAS) from 10/10 before to 8/10 after implementing cryotherapy, splinting technique, and active range of motion (AROM) exercise. Healthcare professionals can enhance the well-being of senior burn victims by incorporating evidence-based techniques and creative approaches in the rehabilitation process.

## Introduction

A burn injury (BI) is damage to the skin or other organic tissues. It is primarily caused by heat but can also result from radiation, radioactivity, electricity, friction, or chemical contact [1]. Burns are a major worldwide issue in terms of public health, being the fourth most prevalent form of injury among civilians, notably after road accidents, falls, and acts of interpersonal violence [2]. More than 90% of fatalities from burns occur in low- and middle-income nations [1,2]. BI stemming from diverse physical or chemical factors is perceived as avoidable injuries. They typically transpire due to accidents at home, increasing high morbidity and mortality rates. These occurrences are particularly prevalent among victims, especially children and the elderly [3]. The World Health Organization (WHO) states that 300,000 individuals pass away each year as a consequence of burns and burn-related factors globally. Burns also impact a larger number of people by causing disabilities and loss of function [3]. The WHO predicts that there are 11 million BIs of several kinds happening universally per annum, with 180,000 causes of death.

The occurrence of BIs varies greatly [2]. Burns to the hands are frequent, despite comprising less than 3% of the body surface area. These injuries can significantly impact an individual's quality of life. The complex anatomical structure of the hand, which comprises numerous tendons, ligaments, muscles, and nerves, presents challenges in both the diagnosis and treatment of such injuries. This intricate network within the hand requires specialized care. Therapy is often multidisciplinary, involving multiple specialties such as surgeons, physiotherapists, and occupational therapists. Early and accurate diagnosis of the depth and severity of burn wounds is paramount for formulating an effective treatment plan. Prioritizing this diagnosis is essential to prevent long-term disability and loss of functionality. Furthermore, burns to the hand can often result in complications such as contractures, which necessitate timely and appropriate intervention. Thus, meticulous wound management is essential. Early rehabilitation must be incorporated into the overall treatment strategy [4].

Complications following burns include swelling and compartment syndrome, which can lead to inadequate blood flow. Hypertrophic scars often develop after BI. The location of the hand injury can significantly affect recovery. A burn to the hand can negatively impact activities of daily living and the functioning of individuals. Depending on the burn's location and severity, it could result in noticeable disfigurement. Functional impairment resulting from burns includes edema and a limited range of motion (ROM). There is an increased risk of contracture deformities and tendon injuries. A study involving 307 victims of BIs at multiple centers found a strong connection between longer therapy duration and reduced burn wound contractures. This was a preliminary study showing a connection between rehabilitation treatments and patient outcomes [5]. Occupational therapy, physical therapy, and mobilization are essential aspects of treatment following BIs. These interventions can significantly lessen joint stiffness and mitigate traumatic myositis ossificans, as well as improve muscle and tendon functionality. Furthermore, exercise can decrease the hypermetabolic response. Exercise therapy should start early, and it is important to begin mobilization immediately upon admission to the hospital. After surgery, it is crucial to minimize any restrictions [6]. Rehabilitation focuses on preventing scars and controlling hypertrophic scars, managing heterotopic ossification, leukoderma, and pruritus. It aims to restore full functional ability, including flexibility, strength, and independent mobility for daily activities. Additionally, it encompasses treatments for complex reconstructive surgeries and strategies to reintegrate individuals into their home and community environments [7]. Recognizing the profound physical and mental effects of burns, it's essential to explore how physiotherapy can aid in enhancing flexibility, minimizing scarring, managing pain, and alleviating anxiety for patients recovering from a 20% second-degree, superficial partial-thickness flame burn post-surgery. This research aims to bridge existing knowledge gaps by offering a comprehensive approach to burn rehabilitation, emphasizing patient care, pain management, mental health, and functional improvement. The study promises to provide valuable insights into improving the understanding and efficacy of treatment methods for this aging population.

## Case presentation

A 71-year-old woman (60 kg, 1.2 m) visited the Physiotherapy Outpatient Department, reporting a burn complication on her right hand, with limited movement in her fourth and fifth fingers for the past two months. Swelling and wound contracture are also present. Two months ago, she suffered burns to her right hand from a gas stove and needed immediate medical care. She was first treated in a nearby hospital, where dead tissue was removed and her burn was covered with dressing. Due to the severity of her injury, she later requested further treatment in a private hospital, where she underwent a split skin grafting procedure to treat the burn damage. In addition to her burn injuries, she has limited mobility in her fourth and fifth fingers, which may affect her hand function and overall quality of life. Over the past five years, she has also been treated for hypertension with a daily dose of 5 mg amlodipine. Additionally, her medical history includes a partial nephrectomy performed three years ago, which indicates an important previous health problem that may affect her current treatment options and recovery from burn injuries. After removing her dressing and stitches from her right hand, she was sent to the Physiotherapy Outpatient Department (OPD) for further treatment. The patient was recovering from a serious BI, with ongoing challenges related to swelling, limited finger mobility, and wound contracture. She moved to rehabilitation physiotherapy to address these problems and improve her functional abilities.

Physical examination

**Figure 1:**
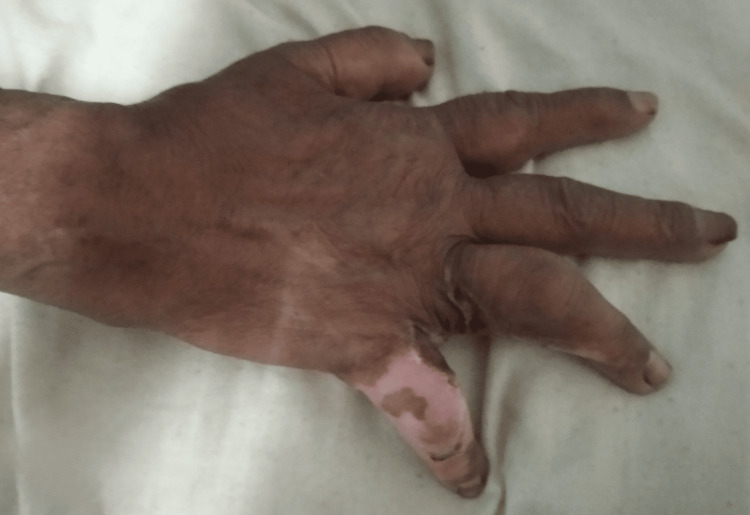
Wound contracture with second-degree burn operated with split skin grafting on the fourth and fifth digits of fingers

The visual analog scale (VAS) pain rating was 10/10, increasing during work and decreasing during rest. The onset of pain was sudden; it was constant, burning, and worse at night. There was a one-inch scar on the fourth and fifth digits and a one- to two-inch scar on the front of the left leg. Inflammation was noted in the right hand and all fingers during the examination. Grade 1 tenderness was found in the fourth and fifth digits, and tightness was observed in the wrist extensors and finger flexors. The Wallace Rule of Nines indicates that 2% of burns have been found, as shown in Figure 1, which shows burn contracture in the hand.

The ROM examination is mentioned in Table 1.

**Table 1:** The range of motion of the MCP joint and IP joint for the left and right hands MCP: Metacarpophalangeal; IP: Interphalangeal

Movement	Left (non-affected)	Right (affected)
At wrist		
Radial	0-20	0-15
Ulnar	0-30	0-15
Flexion	0-80	0-60
Extension	0-70	0-50
At hand (fingers)		
Thumb flexion	0-15	0-10
Thumb extension	0-20	0-10
Thumb abduction	0-70	0-55
MCP flexion	0-90	0-80
MCP extension	0-45	0-35
IP flexion	0-80	0-65
IP extension	0-20	0-10

The examination of MMT for the left and right hands is mentioned in Table 2.

**Table 2:** The aforementioned examination of MMT according to the Oxford Scale was conducted on both the left and right hands PIP: Proximal interphalangeal joint; DIP: Dorsal interphalangeal joint; IP: Interphalangeal joint; MMT: Manual muscle testing

Wrist, hand, and finger (MMT)	Left hand	Right hand (affected)
Wrist extensors	4/5	3/5
Wrist flexors	4/5	3/5
MCP flexors	4/5	2/5
MCP extensors	4/5	2/5
MCP abductors	4/5	2/5
MCP adductors	4/5	2/5
PIP flexors	4/5	2/5
PIP extensors	4/5	2/5
DIP flexors	4/5	2/5
DIP extensors	4/5	2/5

Physiotherapy management

The customized physiotherapy protocol for burn hand injuries in elderly patients, as shown in Table 3, was developed.

**Table 3:** The tailored physiotherapy protocol for elderly burn hand injury patients operated with split skin grafting MET: Muscle energy technique; MCP: Metacarpal phalangeal joint; PIP: Proximal interphalangeal joint; DIP: Dorsal interphalangeal joint; ROM: Range of motion

Goals/aim	Exercises intervention	Repetition/duration
To reduce pain and swelling	Use cryotherapy and elevate the hand to 90 degrees with a pillow placed underneath, elevating the hand above your chest level with a straightforward and efficient method to prevent and reduce swelling, according to Kamolz et al. (2009). Ultrasound should be applied daily for 10 minutes, once [8].	4-5 times a day for 10 minutes
To increase blood circulation and break adhesion formation	Technique for Massaging Scars Massage in the opposite direction to improve blood flow back to the heart, enhance lymphatic flow, move fluid around, and use gentle strokes to boost circulation. Applying static pressure helps decrease swelling in certain areas of the finger and thumb, while kneading helps move the scar and nearby tissue, and skin rolling is used to improve mobility in tissue connections. Pressing the scar to encourage collagen remodeling and rubbing to break up adhesions [9].	3-4 times a day
To reduce edema and manage hypertrophic scars	Pressure garment therapy (PGT) [10]	3 times a day
For ‘claw’ deformity prevention (intrinsic minus position)	Active ROM and Passive ROM exercises [11]	3 times a day
To avoid deconditioning	Mobilization of MCP, DIP, PIP, and wrist joints [12]	3 times a day
To improve hand-functional activities	Innate bending of the metacarpophalangeal joints and inherent extension of the interphalangeal joint, overall grasping (also known as composite bending), upkeep of the spaces between the fingers, and bringing the thumb towards the other fingers.	3-4 times a day
Home exercise program [11]	Exercise No. 1 - Perform by extending your shoulder and elbow fully over your head. Bend your elbow at a 90-degree angle close to your waist, rotating your hand to point towards the ceiling and then towards the floor. Extend your thumb to make contact with the bottom of your pinkie finger.	10 times a day
	Exercise No. 2 - Bend your fingers gently towards your palm, then let them stretch back to the splint.	3 times a day
	Exercise No. 3 - With your opposite hand, slip a finger behind the knuckle in the middle and position your thumb on the fingernail. Carefully move each finger in the direction of your palm.	Repeat 3 times
	Exercise No. 4 - Using your other hand, slide one finger behind the middle knuckle and straighten the top of your finger. This should stretch the front of your finger.	Repeat 3 times
To improve muscle strength and cardiopulmonary endurance	Resistance strength training and aerobic training	3 times per week
To improve functional capacity of the lungs	Breathing exercise - Diaphragmatic breathing exercises and thoracic expansion exercises	10 times each day
To increase ROM and reduce joint stiffness [11]	MET for wrist and 4 and 5 fingers of MCP and PIP joint	3 times a day
Stretching of wrist and finger [12]	Flexors, extensors abductors, and adductors	3 times a day for a 30-second hold
Breathing exercises and visualization technique	For distraction from the painful stimulus of burns	3 times a day

Outcome measures

The pre- and post-outcome measures are presented in Table 4.

**Table 4:** The pre- and post-outcome measures were taken for five weeks VAS: Visual analog scale; DASH: Disabilities of the arm, shoulder, and hand

Outcome	Pre-intervention	Post-intervention
VAS	10/10	8/10
Mayo Wrist Score [13]	10/100	50/100
DASH Score [13]	70/100	50/100
Burn Specific Health Scale - Brief [14]	35/40	25/40
Older People Quality of Life Questionnaire Scale [15]	38	45

## Discussion

This case study highlighted the necessity for a standardized approach to evaluate and treat subjects with burn hand injuries. The data in the study was collected using a goniometer, the Vancouver Scar Scale, the Burn Specific Health Scale - Brief (BSHS-B), and the Older People's Quality of Life (QoL) Scale. The case study findings demonstrated the positive effects of physiotherapy interventions on the burn subject, specifically regarding scar formation improvement, functional ability enhancement, and pain reduction. To create a successful workout plan for senior patients with BIs on their hands from a physiotherapy standpoint, it is crucial to consider this group's specific obstacles and needs. Older adults with BIs tend to have slower wound healing, increased mortality rates, and diminished recovery abilities in comparison to younger patients [7]. Hence, enhancing rehabilitation strategies is essential to improve their functional outcomes and overall health. Sufficient joint mobility is essential for the correct growth, alignment, and formation of bones, muscles, and connective tissues. The study's results were beneficial in enhancing ROM, as seen in Deng et al.'s research, which showed improved joint mobility and shorter hospital stays through physiotherapy mobility training [16].

Studies show that it is essential to have a thorough rehabilitation program in place to help patients recover complete hand function after experiencing burn trauma [16]. Integrating physiotherapy into burn treatment has positively impacted biochemical parameters in major burn patients, highlighting the importance of early rehabilitation interventions [17]. Additionally, research underscores the importance of specialized burn treatment to improve survival rates for elderly individuals with BIs [18]. It is essential to assess the effectiveness of various modalities when creating an exercise protocol. Research has demonstrated that performing ROM exercises while undergoing hydrotherapy can improve hand function and decrease pain levels in burn patients [16]. Innovative methods, like using smart gloves that require active ROM exercises, could offer new possibilities for rehabilitation [17]. Moreover, it is crucial to effectively address pain during physiotherapy to achieve the best results in BI rehabilitation [18]. Sufficient pain control improves patient comfort and encourages participation in rehab, leading to improved recovery. In this research, we reveal that administering the mentioned workout to the individual proved advantageous, with a VAS pain rating decreasing from 10/10 before to 8/10 after implementing cryotherapy, splinting techniques, and active range of motion (AROM) exercises.

Before the mobilization and stretching exercises, Myo's wrist score [13] was 10/100. After completing the exercises, the post score improved to 50/100. The disabilities of the arm, shoulder, and hand (DASH) [13] focused on evaluating the functional movement of the hand, wrist, and shoulder. The pre-assessment score for DASH was 70/100, while the post-assessment score was 50/100. The patient was instructed to perform functional exercises such as wrist supination and pronation, grip strengthening exercises, ball squeezing exercises, and fine and gross motor activities. BSHS-B [14] scored 35/40 in the pre-assessment and 25/40 in the post-assessment, following the protocol mentioned above. Physiotherapy treatment improves the QoL for older individuals by increasing it after being reduced, showing its benefits for the patient on the scale of the older people's QoL questionnaire [15]. The study results indicated that there were positive effects on improving ROM, consistent with Deng et al.'s research, which demonstrated reduced hospital stays and enhanced ROM in various joints through physiotherapy mobility training [16]. This study found that using therapeutic ultrasound helped improve scar formation, confirming the results of a study by Liuzzi et al. that demonstrated the efficacy and safety of therapeutic ultrasound in scar healing [17]. The results of this research indicated that physiotherapy treatments were effective in decreasing anxiety levels among individuals with burns, which aligns with a study by Najafi Ghezeljeh et al. that found music and massage therapy reduced pain and anxiety levels in burn patients [18].

## Conclusions

To summarize, developing a personalized exercise program for elderly people with burn hand injuries necessitates a collaborative effort involving physiotherapy, pain management, and specialized burn care. Healthcare providers can enhance the QoL of elderly burn patients by implementing evidence-based practices and utilizing innovative modalities in their rehabilitation process.
